# Mutational analysis of driver genes with tumor suppressive and oncogenic roles in gastric cancer

**DOI:** 10.7717/peerj.3585

**Published:** 2017-07-17

**Authors:** Tianfang Wang, Yining Liu, Min Zhao

**Affiliations:** 1 School of Science and Engineering, Faculty of Science, Health, Education and Engineering, University of the Sunshine Coast, Maroochydore DC, Australia; 2 The School of Public Health, Institute for Chemical Carcinogenesis, Guangzhou Medical University, Guangzhou, China

**Keywords:** Gastric cancer, Oncogene, Tumor suppressor gene, Micro-RNA, Driver gene

## Abstract

Gastric cancer (GC) is a complex disease with heterogeneous genetic mechanisms. Genomic mutational profiling of gastric cancer not only expands our knowledge about cancer progression at a fundamental genetic level, but also could provide guidance on new treatment decisions, currently based on tumor histology. The fact that precise medicine-based treatment is successful in a subset of tumors indicates the need for better identification of clinically related molecular tumor phenotypes, especially with regard to those driver mutations on tumor suppressor genes (TSGs) and oncogenes (ONGs). We surveyed 313 TSGs and 160 ONGs associated with 48 protein coding and 19 miRNA genes with both TSG and ONG roles. Using public cancer mutational profiles, we confirmed the dual roles of *CDKN1A* and *CDKN1B*. In addition to the widely recognized alterations, we identified another 82 frequently mutated genes in public gastric cancer cohort. In summary, these driver mutation profiles of individual GC will form the basis of personalized treatment of gastric cancer, leading to substantial therapeutic improvements.

## Introduction

Gastric cancer (GC) is the fourth most common cancer worldwide and, although rates have been declining by approximately 2% per year, it is responsible for the second highest rate of cancer-related morbidity and mortality (Bertuccio et al., 2009; Bosetti et al., 2013; Peleteiro et al., 2014). The clinical outcomes for patients with advanced gastric cancer are poor, despite the significant efforts that have been devoted to the development of therapeutic treatments (De Martel, Forman & Plummer, 2013; Karimi et al., 2014). Studies investigating molecular and biochemical changes in GC tissues/cells indicate that the development of GC is a complex process involving function-altering mutations of oncogenes (ONGs) and tumor suppressor genes (TSGs) (Brabek et al., 2010; Gan et al., 2015).

Proto-oncogenes are a group of genes that, when mutated, cause normal cells to become cancerous; the mutated version of a proto-oncogene is called an oncogene (Adamson, 1987; Weinstein & Joe, 2006). Usually, proto-oncogenes encode proteins that stimulate cell division, inhibit cell differentiation, and inhibit cell death, while oncogenes increase production of these proteins and are considered as major molecular targets for anti-cancer drugs (Druker, 2002; Luo, Solimini & Elledge, 2009; Weinstein, 2002). TSGs represent are guardian genes that play important roles in controlling cell growth processes such as cell-cycle checkpoints and inducing apoptosis in normal cells (Macleod, 2000; Weinberg, 1991). In many tumors, TSGs are often inactivated, removing the restriction on cell proliferation and resulting in the progression of tumor cells (Greenblatt et al., 1994; Narla et al., 2001; Tamura, 2006). A number of ONGs and TSGs have been characterized in GC. For example, frequent mutation of *p53* has been observed (Tamura et al., 1991) and *E-cadherin* activation, induced by mutations increase gastric carcinogenesis (Tamura et al., 1996). Promoter hyper-methylation of TSGs has been described in GC including *APC*, *CHFR*, *COX2*, *DAP-kinase*, *DCC*, *E-cadherin*, *GSTP1*, *HRK*, *hMLH1*, *LOX*, *MGMT*, *P14*, *P15*, *P16*, *PTEN*, *RASSF1A*, *RUNX3*, 14-3-3 sigma, *THBS1*, *TIMP-3*, *and TSLC1* (Byun et al., 2001; Fleisher et al., 1999; Honda et al., 2004; Leung et al., 2001; Li et al., 2002; Obata et al., 2003; Sato et al., 2002; Satoh et al., 2002; Suzuki et al., 1999; Tamura et al., 2001; Tamura et al., 2000; Toyota et al., 1999; Tsuchiya et al., 2000).

Based on GC-implicated genes, we utilized an integrative analysis to identify potential TSGs and ONGs in GC. Additional driver gene prediction further prioritized the highly frequently mutated genes in The Cancer Genome Atlas (TCGA) gastric cancer dataset. Our study produced an up-to-date literature-based survey dedicated for TSGs and ONGs in gastric cancer and provides an important resource for large-scale advanced genetic screen and indication for experimental validation. We also identified 48 protein-coding genes and 19 miRNAs with both TSG and ONG roles. Those coding and non-coding dual role drivers primarily function as regulators in cell proliferation, which implies the potential reverse effect on those genes. The adoption of treatments, tailored according to the suppressive or oncogenic functions of these dual role genes, would involve a paradigm shift in cancer therapy but could lead to improvements in treatment.

## Materials & Methods

### Gene list related to gastric cancer

To systematically study the GC-related genes, we downloaded all 1,815 literature-based GC-related genes were downloaded from GCGene for further analysis (Zhao et al., 2016a). The GCGene was constructed by performing an extensive data integration and literature search followed by manual assembly of the data. To provide a more reliable gene list, we also download 683 genes with two or more PubMed abstracts, which represent reliable gene list related from GCGene. The full GC-related gene list provided a basis for integration. By subtraction of those well-studied 683 genes, we also collected 1,132 genes with a single reference, which will help identify unexplored genes and pathways.

### Data source for driver identification in gastric cancer

To identify the driver genes in gastric cancer, we used three bioinformatic databases: (i) TSGene (Zhao et al., 2016b), including 1,207 known human TSGs curated from literatures; (ii) ONGene (Liu, Sun & Zhao, 2016), the ONG list with 803 human genes from 8,849 PubMed abstracts; and (iii) DriverDB 2.0 (Chung et al., 2016), a comprehensive cancer driver genes database constructed by integrating 15 published bioinformatics driver identification algorithms.

For TSGene and ONGene, we downloaded all the genes including protein-coding and non-coding genes from corresponding websites. For DriverDB 2.0, we focused on the TCGA GC dataset and predicted putative driver genes in all the GC samples by using the integrated 15 driver identification tools. To obtain a reliable driver gene list, we required that any driver gene should be supported by at least two tools based on the TCGA mutational data.

### Pathway and mutational analysis

To assess the function of all the identified GC-related driver genes, we conducted functional enrichment tests using the online tool ToppFunc (Chen et al., 2009). ToppFunc adopts a hypergeometric model in order to measure whether an input gene list has a different annotation frequency to the one that would occur randomly. We conducted chromosome cytoband-based enrichment analysis to identify the genomic regions where the input genes were significantly enriched using all the genes in these regions as background. Similar processes were used to identify enriched gene ontology terms, KEGG and wiki pathways. In these enrichment analyses, all the human genes in ToppFunc were used as background to calculate statistical significance. In addition, the Benjamini–Hochberg method was implemented in the ToppFunc to further exclude false negative results. Finally, a *p*-value <0.01 was adopted as the cutoff for enriched pathways in KEGG and gene ontology (biological process) and we only considered those representative pathways with two or more genes. For the enrichment analysis on miRNAs, we adopted an online server miEAA (Watanabe et al., 2007). MiEAA offers both over-representation analysis and set enrichment analysis, which is similar to gene set enrichment analysis implemented in ToppFunc.

Throughout the study, the GC-related mutational analyses were conducted using the cBio portal (Cerami et al., 2012). We selected the Stomach Adenocarcinoma (TCGA) dataset. In total, there are 393 tumor samples with single nucleotide mutations, INDELs, or copy number variation (CNV) data. Abnormal gene expression and protein expression were not included. For the DNA copy-number data, the putative discrete values were calculated for all genes, e.g.,  “deeply deleted” or “amplified”. For single-nucleotide variations and INDELs, we excluded those mutations without any functional effect, such as synonymous mutations.

## Results & Discussion

### Identification of dual role protein-coding genes as TSGs and ONGs in gastric cancer

**Figure 1:**
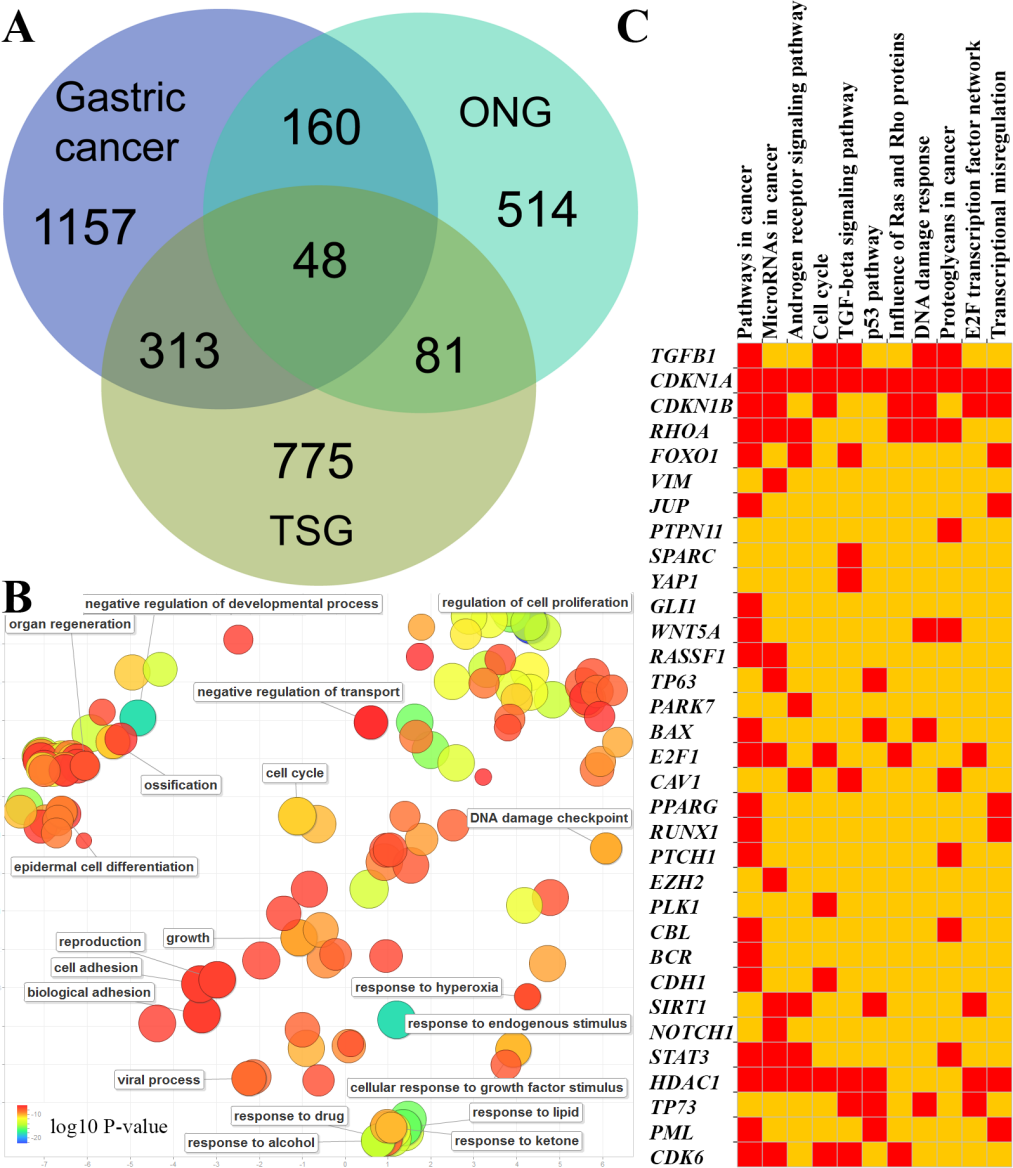
GC-implicated genes with tumor suppressor and oncogene roles. (A) The shared GC-implicated genes with tumor suppressor and oncogene roles in GCGene database. (B) The statistically over-represented gene ontology terms for 48 genes with both tumor suppressor and oncogene roles. (C) The profile of enriched KEGG pathways for 48 genes with both tumor suppressor and oncogene roles.

To survey how many TSGs and ONGs are involved in the development of gastric cancers, we used the data from three large-scale literature mining databases. The GCGene is the database developed to curate the gastric cancer-related genes from literature. The TSGene and ONGene were the databases to collect known critical cancer genes with literature. By overlapping the protein-coding genes from GCGene to TSGene and ONGene, we identified 313 TSGs,160 ONGs (Fig. 1A) and 48 dual role genes with both TSGs and ONGs effects in other cancers (Table S1).

Many genes could function as both TSG and ONG depending on the cancer type, stage of development, or interaction partners (Zhao et al., 2016b). For example, as an oncogene, *SIRT1* can promote cancer progression by negative control of the TGF-beta signaling pathway (Lamouille & Derynck, 2009). However, *SIRT1* can also interact with promyelocytic leukemia protein to express its tumor suppressor property by stabilizing *TP53* and inducing cell senescence. To provide a global functional distribution of the 48 dual role genes in GC, we performed a functional enrichment analysis of gene ontology, KEGG pathway, genomic location, and protein family (Figs. 1B–1C, Table S2). We found 35 genes are active regulators in cell proliferation (GO:0042127, corrected *P*-value = 2.89E–22). More interesting, there are 32 genes involving in “response to endogenous stimulus” (GO:0009719, corrected *P*-value = 6.70E–18). Consistently, the subcellular localization also mainly group into nuclear chromosome (11 genes, GO:0000228, corrected *P*-value = 9.13E–06) and the plasma membrane region (12 genes, GO:0098590, corrected *P*-value = 1.57E–04). Those genes located on chromosome or chromatin are mainly from transcription factor complex. For example, we found five p53-like transcription factors: *RUNX1*, *RUNX3*, *STAT3*, *TP63*, and *TP73* (InterPro domain IPR008967, corrected *P*-value = 3.65E–05). By mapping to the pathways (Fig. 1C), we were able to locate those genes in the critical cancer pathways. The dual role genes are associated with cancer pathways (corrected *P*-value = 5.75E–20), microRNA (corrected *P*-value = 2.72E–10) and transcriptional regulation (corrected *P*-value = 9.64E-07). Cell cycle (corrected *P*-value = 1.22E–08) and response to DNA damage (corrected *P*-value = 1.24E–07) may be controlled by the dual role genes which are also competing in the androgen receptor (corrected *P*-value = 3.74E–09), TGF-beta (corrected *P*-value = 1.84E–08) and p53 signaling (corrected *P*-value = 4.60E–08) pathways. In summary, our integrative analysis revealed that those genes with both TSG and ONG roles may group into two main functional clusters in gastric cancer: transcriptional regulation inside nuclear, and response to endogenous signals in plasma membrane region. The competition of these genes in critical pathways, such as the cell cycle p53 signaling pathway, may be critical for GC progression.

**Figure 2:**
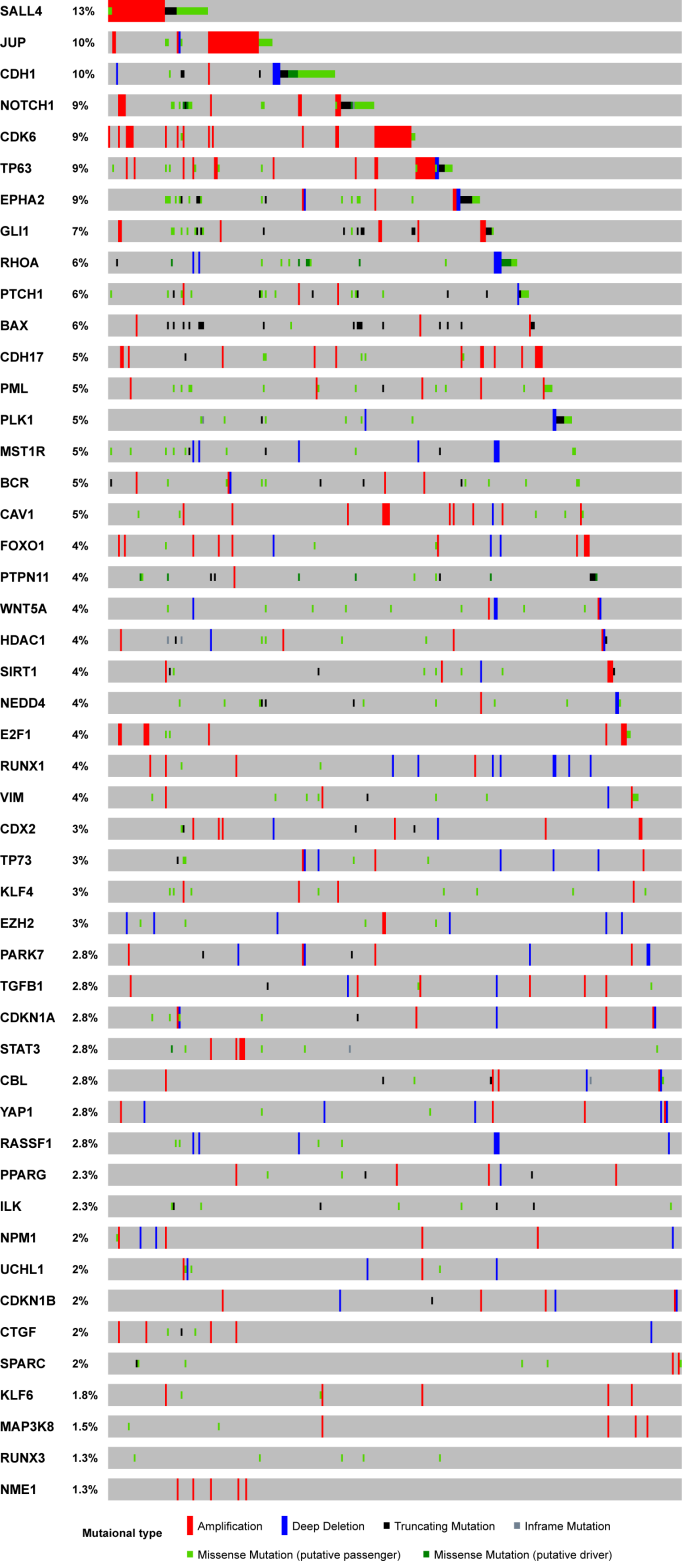
Mutational profile for the 48 genes with both tumor suppressor and oncogenes roles. The sample-based mutational profile for the 48 genes with both tumor suppressor and oncogenes roles in TCGA stomach dataset. Each gene is depicted in each row across multiple samples (each sample for a single column).

The majority of genes are unique to some pathways (Fig. 1C). However, several genes, including *CDKN1A* and *CDKN1B,* are involved in multiple oncogenic pathways (Fig. 1C). Due to their dual functions in these critical oncogenic pathways, we need to be cautious about drawing conclusions concerning their effects on GC cells. By overlapping those genes with TCGA somatic mutation data, we investigated the potential functions of the 48 genes with dual roles in TCGA GC samples. A few genes did not have dual roles according to their mutational pattern (Fig. 2). For example, *SALL4*, *JUP*, *NOTCH1* and *CDK6* are all with frequent amplification in multiple cancer samples, which may indicate their oncogenic roles in GC. In contrast, more copy number deletions were observed in *CDH1*, *RHOA*, *PLK1*, and *MST1R*, which may imply a TSG role in GC. For the two genes with broad effects, *CDKN1A* and *CDKN1B*, both gene copy gain and loss were found, which means they may have dual roles in GC. The mutational pattern also confirmed some of the genes, such as *CDKN1A* and *CDKN1B,* may have dual roles in the TCGA GC cohort.

### The functional and mutational features of protein-coding driver TSGs and ONGs in TCGA GC cohort

**Figure 3:**
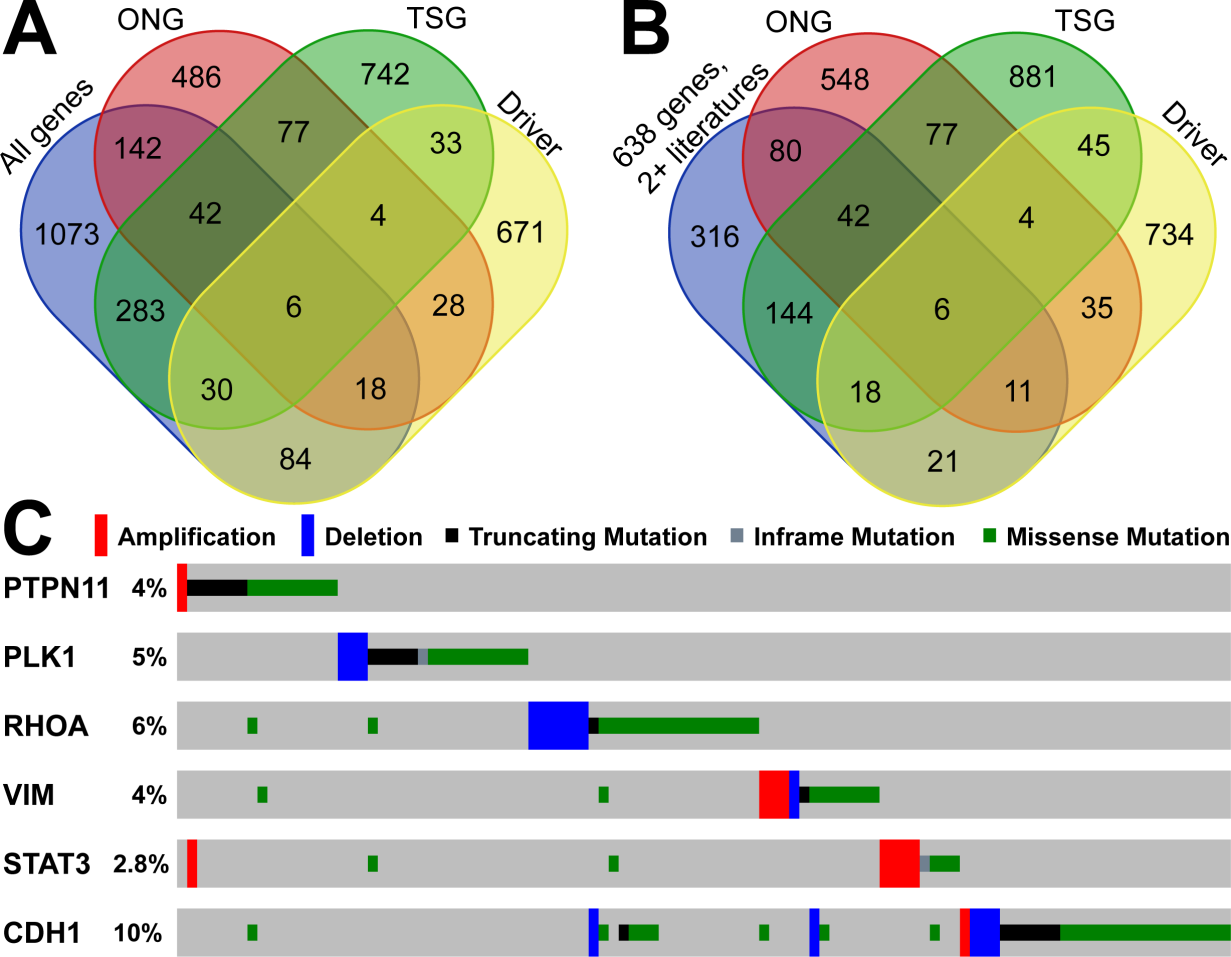
Overlapping analysis of GC-implicated genes, tumor suppressors, oncogenes and predicted driver genes. (A) The overlapping of coding GC-implicated genes, tumor suppressors, oncogenes and predicted driver genes from Driver DB 2.0. (B) The overlapping of reliable GC-implicated genes with two or more literature evidence, tumor suppressors, oncogenes and predicted driver genes from Driver DB 2.0. (C) The sample-based mutational profile for the six driver genes with both tumor suppressor and oncogenes roles in TCGA stomach dataset.

To explore the driver TSGs and ONGs, we utilized 15 driver mutational detection tools to identify the protein-coding drivers in the TCGA GC cohort. For a driver gene, we required two positive results from two or more driver detection tools. In total, we found 874 genes with driver mutations in the TCGA data. By overlapping with the gastric cancer-related genes in GCGene, we identified those driver genes with and without TSG and ONG roles. We found 30, 18, 6, and 84 driver genes as TSGs, ONGs, dual role, and non-TSG-ONG respectively (Fig. 3A). We considered that those 138 driver genes were well-studied in gastric cancer. However, based on the number of literature evidence in GCGene database, we found there are only 56 driver genes with two or more literature evidences (Fig. 3B). All the six genes with dual roles in cancers are supported by at least two references, which confirmed their important roles in GC. By using TCGA GC mutation data, we explored the mutational frequency of the six genes (Fig. 3C). In total, there were 105 instances with at least one somatic mutation (copy number variation included) in 393 sequenced patients (27%). Some of the six genes had only one function based on the mutational pattern. For example, *STAT3* is amplified in the majority of mutated samples suggesting that the activation of STAT3 signaling genes supports GC cell survival (Kanda et al., 2004). Similarly, *PLK1* and *RHOA* were deleted in a number of patients and behave more like TSGs. However, the remaining three genes (*PTPN11*, *VIM*, and *CDH1*) are more likely to have dual roles with both mutations for gain-of-function and loss-of-function.

**Figure 4:**
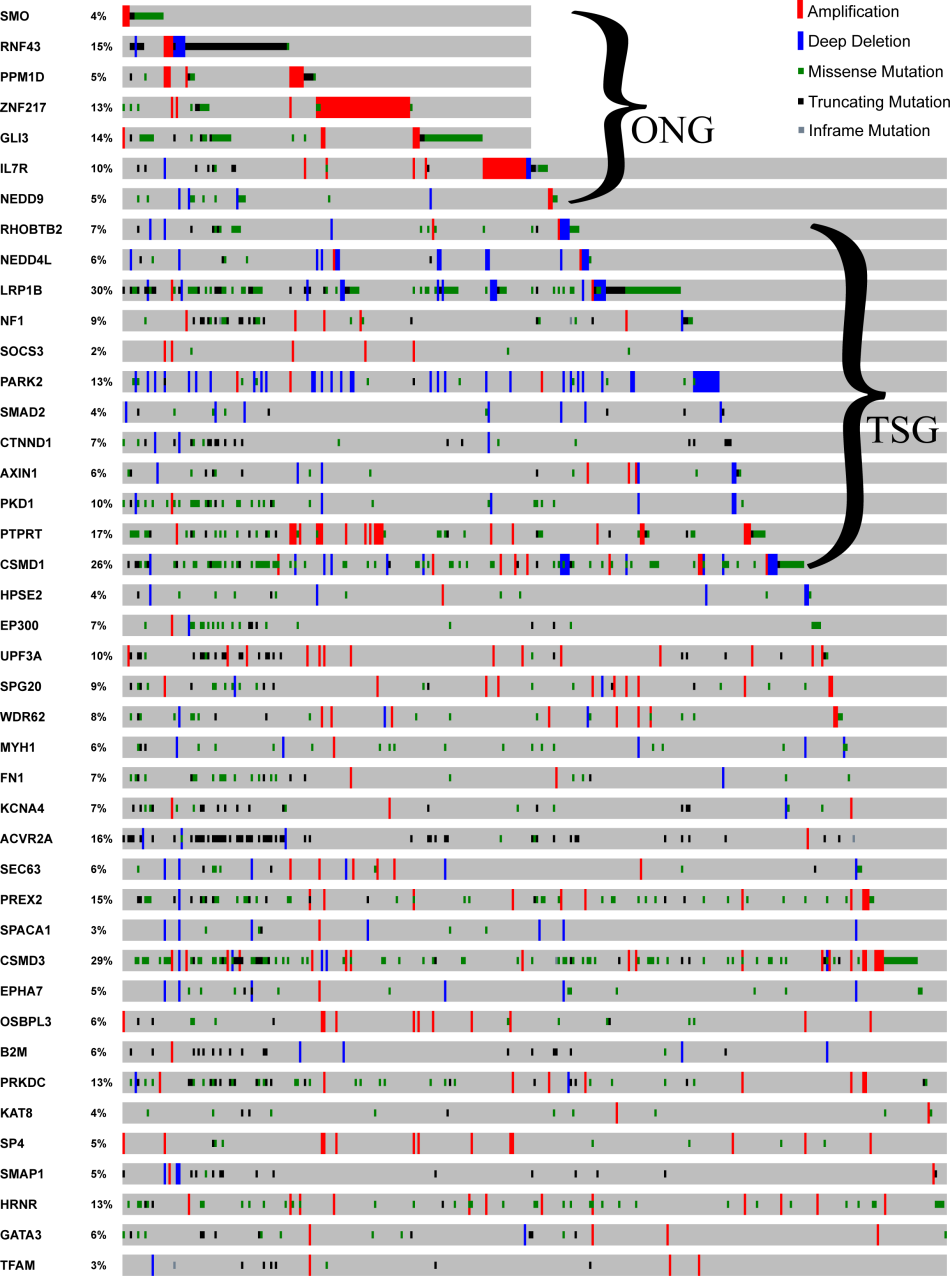
Mutational profile for the 82 driver genes. The sample-based mutational profile for the 82 driver genes with single literature evidence. Each gene is depicted in each row across multiple samples (each sample for a single column).

To discover some novel driver genes not extensively studied in GC, we focused on 82 predicted driver genes with one reference in the GCGene database (Figs. 3A, 3B). There were 12 TSG and 7 ONG driver genes associated with a single PubMed abstract. Among the 82 non-TSG-ONG driver genes (Fig. 3A), 63 are not well studied in GC (Fig. 3B and Table S3). To validate the functions of these genes in GC it is important to check over the mutational frequency, we investigated the 82 putative drivers in the TCGA GC mutational data (Fig. 4). All of the seven ONG driver genes are highly mutated with a highest mutational frequency of 14% on *GLI3* and the lowest mutational frequency 4% on *SMO*. Only the ONG *NEDD9* had sporadic deletions, which is not consistent with its oncogenic role. For 12 of the TSG driver genes, we found sporadic amplifications on *AXIN1*, *CSMD1*, *LRP1B*, *NEDD4L*, *NF1*, *PARK2*, and *RHOBTB2*. Furthermore, two of the TSG driver genes (*SOCS3*, *PTPRT)* have concordant amplifications which implies a ONG role in the TCGA GC cohort. For the remaining driver genes, which have no TSG and ONG functions, the majority have both amplifications and deep deletions in multiple cancer samples.

### MicroRNA TSGs and OGCs in gastric cancer

**Figure 5:**
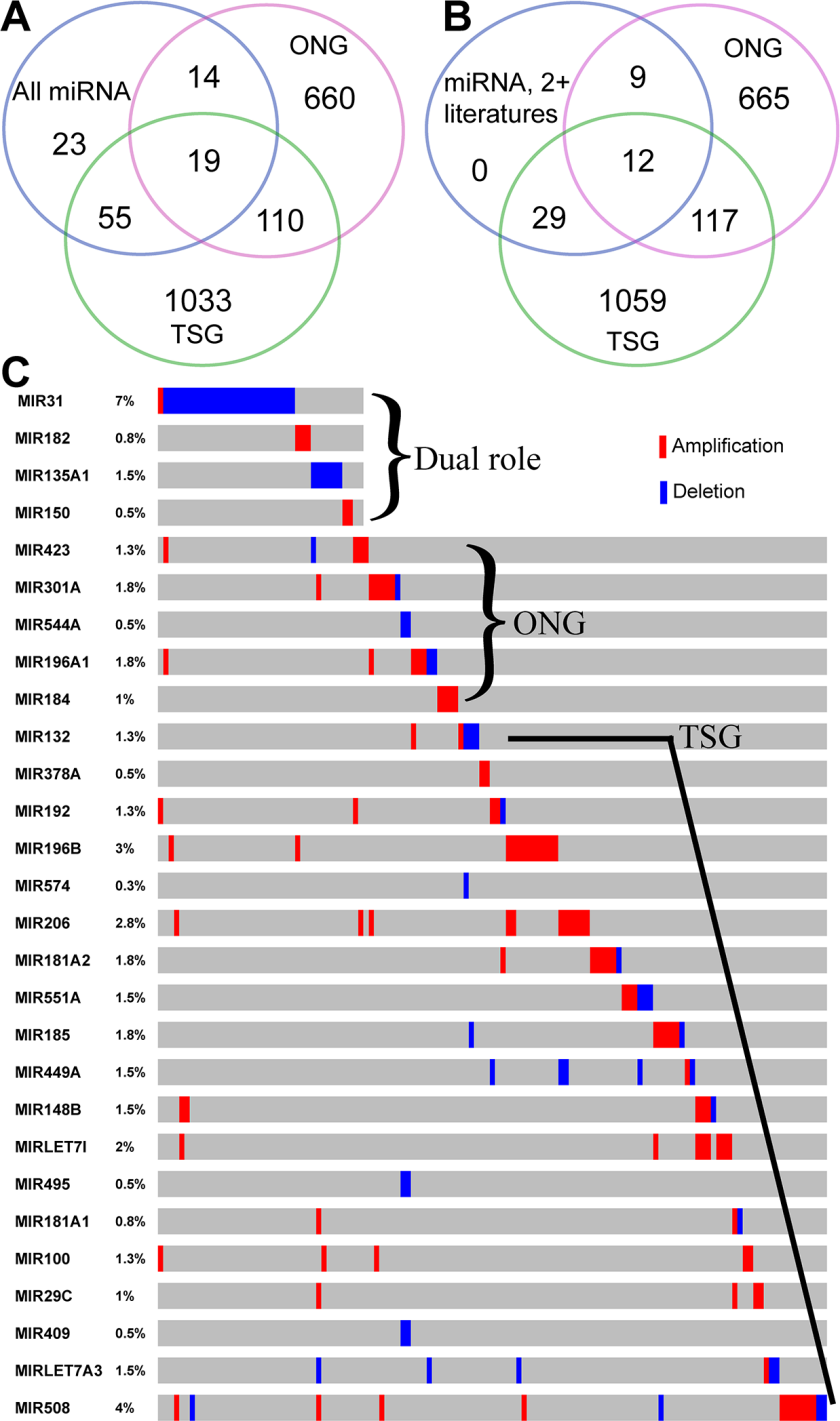
Overlapping analysis of GC-implicated miRNAs, tumor suppressors, and oncogenes. (A) The overlapping of GC-implicated miRNAs, tumor suppressors, and oncogenes. (B) The overlapping of reliable GC-implicated miRNAs with two or more literature evidence, tumor suppressors, and oncogenes. (C) The sample-based mutational profile for the four dual role, five oncogenic, and 19 tumor suppressive miRNAs with single literature evidence (each sample for a single column).

Recent studies have reported that some micro-RNAs (miR), single-stranded, small noncoding RNA genes, can function as TSGs and ONGs. Evidence from our GCGene shows that there are 111 miRNAs related to GC. By intersecting with those curated TSG and ONG miRNAs, we found 55 TSGs, 14 ONGs and 19 dual role miRNAs (Fig. 5A). Among these genes, there are 29 TSGs, 9 ONGs and 12 dual role miRNAs with two or more references in GC (Fig. 5B). Some of the dual role miRNAs are confirmed as having dual roles in gastric cancer. For example, *miR-223* is overexpressed in metastatic GC cells and stimulates non-metastatic GC cells migration and invasion by directly targeting its 3′-untranslated regions of *EPB41L3* (Li et al., 2011). In addition, *miR-223* functions as an oncogene in human GC by targeting *FBXW7/hCdc4* (Li et al., 2012) and targets oncogene *STMN1* (Kang et al., 2012). However, the majority of the miRNAs with dual roles in other cancers are not associated with GC. For instance, *miR-335* was only reported as TSGs to target *Bcl-w* and specificity protein 1 (Xu et al., 2012).

By running an enrichment analysis on the 19 identified dual role miRNAs using miEAA (Watanabe et al., 2007), we confirmed that these miRNAs are enriched in various cancers (Table 1). Among the 19, four (miR-20a, miR-18a, miR-16-1, miR-17) are located on chromosome 13 (Corrected *P*-value = 0.00376467) and three of these belong to the miR-17 family. Although the mir-17/92 cluster are known as oncogenes, recent studies suggest that their dual roles depend on the targeting genes (Xiang & Wu, 2010).

**Table 1:** Enrichment analysis of dual role miRNAs. The enrichment of 19 dual role miRNAs identified in different cancers.

CANCERs	*P*-value	miRNAs/precursors
Neoplasms	1.373E−06	miR-31; miR-20A; miR-18A; miR-149; miR-34B; miR-200C; miR-27A; miR-182; miR-7-1; miR-335; miR-34A; miR-155; miR-16-1; miR-17; miR-150
Stomach Neoplasms	1.373E−06	miR-31; miR-20A; miR-18A; miR-149; miR-34B; miR-223; miR-200C; miR-27A; miR-182; miR-7-1; miR-335; miR-34A; miR-155; miR-16-1; miR-17; miR-107; miR-150
Colorectal Neoplasms	1.66E−06	miR-31; miR-20A; miR-18A; miR-149; miR-34B; miR-200C; miR-27A; miR-182; miR-7-1; miR-335; miR-34A; miR-155; miR-16-1; miR-17; miR-107; miR-150
Pancreatic Neoplasms	1.878E−06	miR-31; miR-20A; miR-18A; miR-34B; miR-223; miR-200C; miR-27A; miR-182; miR-34A; miR-155; miR-16-1; miR-17; miR-107; miR-150
Glioma	5.232E−06	miR-31; miR-20A; miR-18A; miR-149; miR-27A; miR-182; miR-7-1; miR-335; miR-34A; miR-16-1; miR-17; miR-107
Lung Neoplasms	5.232E−06	miR-31; miR-20A; miR-18A; miR-34B; miR-223; miR-200C; miR-27A; miR-182; miR-7-1; miR-335; miR-34A; miR-155; miR-17; miR-107; miR-150
Urinary Bladder Neoplasms	5.232E−06	miR-31; miR-20A; miR-18A; miR-149; miR-34B; miR-200C; miR-27A; miR-182; miR-7-1; miR-34A; miR-155; miR-17; miR-107
Leukemia, Lymphocytic, Chronic, B-Cell	3.762E−05	miR-20A; miR-18A; miR-34B; miR-223; miR-34A; miR-155; miR-16-1; miR-17; miR-107
Ovarian Neoplasms	4.527E−05	miR-31; miR-20A; miR-18A; miR-34B; miR-223; miR-200C; miR-27A; miR-182; miR-335; miR-34A; miR-155; miR-16-1; miR-17
Leukemia	7.58E−05	miR-31; miR-20A; miR-18A; miR-27A; miR-34A; miR-16-1; miR-17; miR-150
Adenocarcinoma	7.627E−05	miR-31; miR-20A; miR-34B; miR-223; miR-200C; miR-182; miR-155; miR-16-1; miR-17
Carcinoma, Non-Small-Cell Lung	7.627E−05	miR-149; miR-34B; miR-223; miR-200C; miR-27A; miR-182; miR-7-1; miR-34A; miR-155; miR-16-1; miR-17; miR-150
Carcinoma, Squamous Cell	8.583E−05	miR-31; miR-20A; miR-18A; miR-34B; miR-223; miR-200C; miR-27A; miR-182; miR-34A; miR-155; miR-16-1

By subtracting those genes shown in Fig. 5B from those in Fig. 5A, we found there are seven dual role miRNAs with single literature evidence in GCGene, which may warrant further investigation. For example, the *miR-150* functioned as ONG to target a TSG *EGR2* (Wu et al., 2010) but there is no information concerning its TSG role in gastric cancer. By overlapping to TCGA CNV data, we found there are four miRNAs with copy numberchanges: *miR-31*, *miR-182*, *miR-135a-1*, and *miR-150*. We found that *miR-31* has both amplification and deletion functions in different tumor samples and this suggests dual roles in gastric cancer. The *miR-182* and *miR-150* only have amplifications but *miR-135a-1* was all deleted in 1.5% TCGA GC cohort. In summary, we found several potential dual role miRNAs in GC with different CNV pattern.

As shown in Figs. 5A and 5B, there are 55 gastric cancer-related miRNAs with TSG functions. A few miRNAs have been suggested as potential TSGs *via* suppressing different oncogenes. For instance, the *miR-148a* (Zheng et al., 2011), *miR-195* and *miR-375* (Ding et al., 2010) may function as TSG in GC, and theirs anti-oncogenic activity may involve the direct targeting and inhibition of ONG *ROCK1*, *CDK6*, and *JAK2.* Some miRNAs may not function in cancer development, but cancer metastasis original from GC tissues. The interaction between *Robo1* on the *Slit2Slit-Robo1* pathway triggers tumor metastasis of GC, which can be suppressed by *miR-218* (Tie et al., 2010). By subtracting those genes shown in Fig. 5B from Fig. 5A, we found there are 26 miRNA TSGs with single literature evidence in GCGene. Examples include *miR-486* (targeting *OLFM4*) (Oh et al., 2011), *miR-378* (suppressing *VEGF*) (Deng et al., 2013), *and miR-101* (targeting *EZH2*, *Cox-2*, *Mcl-1* and *Fos*) (Wang et al., 2010b). Among the 26, there are 19 miRNAs with CNVs in TCGA GC cohort. For example, the *miR-148b* could have a TSG role by targeting *CCKBR* (Song et al., 2011), which was mutated in 1.5% of 393 TCGA patients. Although most of these CNVs for *miR-148b* are deletions, the amplifications were still observed in a few samples. A few of the miRNA TSGs were amplified in all the samples with mutations, such as *miR-196b*, *miR-206, miR-let-7i, miR-100, and miR-29c*, which either implies the potential oncogenic role or those amplifications may not have dosage effects on these miRNAs.

In general, miRNA ONGs are located in the amplified regions in human cancers and tend to cleave target mRNAs more frequently (Wang et al., 2010a). For example, the *miR-21* (Zhang et al., 2012), negatively regulates the tumor suppressors *PTEN* which, in-turn, promote gastric tumor proliferation and invasion. All five ONGs with a single study recorded in GCGene have more amplifications than deletions in the TCGA cohort (Fig. 5C); this confirms their critical oncogenic roles in GC.

## Conclusions

A better understanding of the molecular drivers and pathways of tumor formation has led to the development of targeted agents. In this study, we performed a systematic evaluation of cancer driver genes in GC by integrating literature and mutational data. We identified 313 TSGs and 160 ONGs implicated in GC. By applying driver mutation identification tools, we reduced the gene list to 30 TSGs and 18 ONGs.

As ONGs and TSGs normally perform their cellular functions jointly, different mechanisms have been conceived behind this experimentally based on one or only a few ONGs and TSGs, though controversies remain when considering multiple ONGs and TSGs at a time. Recent investigations have used bioinformatics analysis to compare the mutation patterns and network properties of ONGs and TSGs of different cancers. Distinct regulatory patterns of TSGs and ONGs by transcription factors have been found in ovarian cancer, which competitively acts upon apoptosis and the ErbB signalling pathway (Zhao, Sun & Zhao, 2012). The TSG and ONG miRNAs show distinct patterns in function, evolutionary rate, expression, chromosome distribution, molecule size, free energy, transcription factors, and targets, suggested by a large-scale survey of human miRNA (Wang et al., 2010a). However, we identified 48 coding genes and 19 miRNAs with both TSG and OCG roles. According to the mutation data, some of these genes may have only a single function , in contrast with their role in other cancer types. Interestingly, a few of the genes have mixed mutational patterns with both gain-of-function and loss-of-function. For the first time, we provide the dual role gene list in GC to support further large-scale genetic screen and our systematic evaluation provides a blueprint for the interplay of TSGs and ONGs in GC.

##  Supplemental Information

10.7717/peerj.3585/supp-1 Table S1 The 48 genes with both oncogenic and tumor suppressive roles

10.7717/peerj.3585/supp-2 Table S2 The functional enrichment analysis of 48 genes with dual roles

10.7717/peerj.3585/supp-3 Table S3 The 82 coding driver genes with single literature evidence

## Additional Information and Declarations

### Data Availability

The following information was supplied regarding data availability: The raw data has been supplied as a Supplementary File.
